# Effectiveness of Ultraviolet Radiation and Disinfectant Wipes in Reducing the Microbial Contamination of Mobile Phones in a Tertiary Care Hospital

**DOI:** 10.7759/cureus.64782

**Published:** 2024-07-17

**Authors:** Arnabjyoti De, Barathane Datchanamurthy, Valentina Y, Namrata Bhosale, Senkadhirdasan Dakshinamurthy

**Affiliations:** 1 Medicine, Mahatma Gandhi Medical College and Research Institute, Puducherry, IND; 2 Pharmacology, Mahatma Gandhi Medical College and Research Institute, Puducherry, IND; 3 Microbiology, Mahatma Gandhi Medical College and Research Institute, Puducherry, IND; 4 Community and Family Medicine, Mahatma Gandhi Medical College and Research Institute, Puducherry, IND

**Keywords:** cross-sectional study, hospital acquired infection, bacterial colony, mobile phones, health care professionals, microbial contamination, disinfectant wipes, ultraviolet radiation

## Abstract

Background

As mobile phones act as a potential source of microbial contamination, particularly in a hospital environment, the effectiveness of two most debated interventions namely ultraviolet radiation and disinfectant wipes in reducing the microbial contamination of mobile phones is compared.

Objective

To screen the mobile phones of healthcare personnel for the presence of microorganisms and to compare the effectiveness of ultraviolet radiation and disinfectant wipes in reducing microbial contamination.

Methods and materials

Pre-intervention and post-intervention swabs were collected before and after the use of each intervention respectively using 56 samples and cultured for growth in nutrient agar. Agar plates are subjected to quantitative analysis using bacterial colony count to reflect the efficacy of the specific intervention used. The data collected was entered in Microsoft Excel (Microsoft^®^ Corp., Redmond, WA, USA) and analysis was done using standard statistical packages.

Results

While comparing the pre-intervention bacterial load with the post-intervention load, post-intervention bacterial contamination in terms of colony-forming units/CFU has drastically reduced after both interventions, which is validated by statistical significance. However, it was observed participants using disinfectant wipes as intervention had 2.07 times higher chance of having a low bacterial load which wasn’t statistically significant.

Conclusion

Our study shows that with the use of any intervention from the above-mentioned interventions, bacterial load or bacterial contamination can be reduced significantly, thus pointing out that both ultraviolet radiation and disinfectant wipes are effective in reducing contamination of mobile phones. It was also found that male doctors have more bacterial load than females, which can be minimized by effectively changing behavioral habits.

## Introduction

Today mobile phones have become one of the most essential accessories for both personal and professional life [1]. A square inch of a mobile phone screen contains ten thousand microbes, which is significantly more than the sole of a shoe or a door handle [2]. ​The consistent heat generated by phones creates a breeding ground for the colonization of microorganisms [3]. The regular use of mobile phones makes them a potential source for the transmission of microorganisms that cause various diseases [4]. Healthcare professionals (HCPs) use mobile phones in hospital halls, laboratories, intensive care units, and operating rooms [5]. Moreover, mobile phones are used routinely all day long and the same phones are used outside the hospital playing a possible role in spreading infections to the outside community [5]. During every phone call the mobile phone comes into close contact with contaminated human body areas with hands to hands, and hands to other areas like mouth, nose, and ears, which may result in colonization of potential pathogens present on the human skin, on the mobile phones [6]. Therefore, HCP mobile phones may facilitate the transmission of bacterial isolates from one patient to another in different hospital wards and play an important role in the transmission of hospital-acquired infections (HAI) [6,7]. The widespread use of mobile phones among HCPs is a matter of controversy. In emergency situations, HCPs can seek urgent help from their superiors and colleagues with the help of mobile phones [8]. Another point of view argues that, if mobile phones are used carelessly in surgical wards or intensive care units (ICU), they may act as a source of infection to patients [9]. In order for mobile phones to be successfully used in a clinical setting, appropriate and effective cleaning must be demonstrated. There are a few methods available for the disinfection of mobile phones. Antibacterial wipes for mobile phones are simple and time-saving but have the disadvantage of corroding the protective coating on the glass screen [10]. A newer and innovative method of disinfection of mobile phones is by use of ultraviolet (UV) radiation [11]. Previous literature in our setting has identified different pathogens in mobile phones [12-14] but there is a dearth of studies assessing the effectiveness of using any disinfection technique in reducing the microbial contamination of mobile phones.

## Materials and methods

It is a facility-based cross-sectional study in a 250-plus-bed tertiary care hospital in Puducherry, India. The study involves mobile phones of healthcare professionals (HCPs) - doctors, nurses, and other healthcare workers - who are actively involved in patient care in a tertiary care hospital in Puducherry. The inclusion criteria include healthcare professionals who are actively involved in patient care in intensive care units (ICU), surgical wards and OPD, operation theatres (OT), and emergency departments and who are using smartphones. The exclusion criteria include healthcare professionals working in special wards, non-surgical OPDs, and medical college, using smartphones with physical flaws (for example, a cracked screen), whose mobile phones were purchased less than a month ago, and who had keypad mobile phones (including touchscreen smartphones with keypad).

Sampling procedure

Purposive Sampling

The required number of HCPs was purposely selected from intensive care units, surgical wards and OPDs, operation theatres and emergency departments.

Sample Size

The sample size was calculated using Open Epi version 3.01. A minimum sample size of 28 mobile phones of HCPs was required for the study, considering the prevalence rate of microbial contamination of mobile phones among HCPs as 87.3%, the population size of 2000, relative precision of 15%, and confidence level as 95% (5% alpha error) and 10% attrition rate. For checking the effectiveness of each intervention (ultraviolet radiation/disinfectant wipes), at least 28 mobile phones meeting the inclusion and exclusion criteria were needed.

Ethical Issues

**Figure 1:**
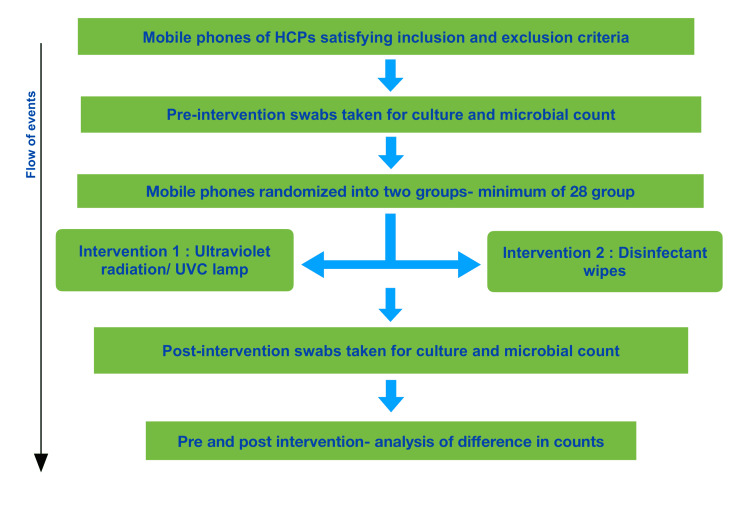
Flow chart showing the progression of events from selection of mobile phones, pre-intervention swab collection, randomization, intervention, post-intervention swab collection and analysis of microbial counts.

The study involved the mobile phones of healthcare professionals in Puducherry. Necessary permissions were sought from the institute management. Confidentiality and anonymity were maintained and the data was used for research purposes only. Institutional Research Committee and Institutional Human Ethics Committee (IHEC) approval was granted before starting the research (Certificate of approval for waiver) (Figure 1).

The described research process was carried out only with personal protective equipment (sterile latex gloves, N95 mask, and face shield). The participant was given a participant information sheet which he had to read and if there were any doubts, those would be clarified. After this procedure, written informed consent was obtained from the participant. After obtaining written informed consent from HCPs, the interview schedule was conducted. A questionnaire with quantitative and qualitative data focusing on the usage of mobile phones, cleaning techniques followed, hygiene practices, etc., which involves the analysis of the behavior of particular HCP using mobile phones, was collected and data was presented using appropriate statistical illustrations. Contact details of the participants involved were collected digitally using the Epicollect5 mobile application for giving back the mobile phones to the appropriate person and the same details were not used in the report for maintaining confidentiality. The mobile phones were transported in sterile zip-lock bags and within the protective containers. Sterile cotton swab (pre-intervention swab) sticks made wet slightly with physiological saline were rotated on the surface and the back of the HCP mobile phone, including the touch screen, and both sides of the phone. After the collection of the swab, samples (mobile phones) were randomized into two groups using Block randomization by computer-generated sequence. Subsequently, the entire mobile phones were disinfected using one of the two decontamination techniques: Disinfectant wipes with 70% isopropyl alcohol and ultraviolet radiation (UVC lamp). To achieve decontamination, two different methods of intervention were used and subsequently termed intervention 1 and intervention 2. Mobile phones were placed in laminar airflow chambers equipped with UVC lamps for 5-10 minutes which is the Intervention-1 and disposable, commercially available disinfectant wipes with active ingredients of 70% isopropyl alcohol were applied on mobile phones for 40 seconds (minimum) which is the Intervention-2. After the intervention, the post-intervention swab was collected. Collected samples were immediately cultured on nutrient agar plates using sterile swab sticks. ​The mobile phones were returned to the participant in sterile zip lock bags at the end of these processes. ​Cultured plates were transported to the laboratory and kept for overnight incubation at 37 degrees Celsius. The media was examined for any growth at 24 hours. The agar plates along with obtained growth were subjected to quantitative analysis using the standard method of bacterial colony count (colony forming units/CFU) which is a measure of bacterial load and thus reflects the efficacy of the intervention used. The data collected was entered in Microsoft Excel (Microsoft® Corp., Redmond, WA, USA) and analysis was done using standard statistical packages.

Statistical methods

The data collected was entered in Microsoft Excel and analysis was done using standard statistical packages. The qualitative variables are summarized using frequency and proportion. The quantitative data is summarized using Mean SD and median, IQR (Interquartile Range). The distribution of data was checked and they are not normally distributed, so a non-parametric test (Wilcoxon sign test) was used for comparing the pre- and post-intervention of ultraviolet (UV) and disinfectant (DT), whereas the Mann-Whitney test was used to compare the UV and DT group. p<0.05 is considered as statistically significant.

## Results

It is a cross-sectional study that was done to identify the efficacy of two decontamination techniques, i.e. ultraviolet radiation (intervention 1) and disinfectant wipes (intervention 2), in reducing the microbial contamination of mobile phones in a tertiary care hospital. The assessment was done by comparing the difference in number of colony-forming units present between pre-intervention and post-intervention. Table 1 shows that the majority (89.3%) of healthcare professionals (HCPs) use their mobile phones in the hospital. Males constituted 58.9% of the total sample. Among HCPs, 76.8% are doctors, 14.3% are nurses and 8.9% are other healthcare workers. A total of 85.71% of HCPs use their phones in the home, 83.92% use them in the hospital, half of the HCPs use them in the restroom and 32.14% use their phones in the patient’s room.

**Table 1:** Descriptive table shows the survey questions

Variable	Sub Group	n=56	%
Gender	Female	23	41.1%
	Male	33	58.9%
Occupation	Doctor	43	76.8%
	Nurses	8	14.3%
	Other Healthcare Workers	5	8.9%
Mobile phone users in hospital	No	6	10.7%
	Yes	50	89.3%
Location of usage	Home	48	85.71%
	Hospital	47	83.92%
	Restroom	28	50%
	Patients Room	18	32.14%
Frequency of cleaning the mobile	1x/day	9	16.1%
	More Than 1x/Day	2	3.57%
	Never	11	19.6%
	Occasionally	19	33.9%
	Rarely	15	26.8%
Cleaning agent	"Others (Clorox, lens cleaning solution, soap water)"	12	21.4%
	Alcohol	12	21.4%
	Dry Wipe	32	57.1%
Mobile phone cleaned before	1-24 hours	8	14.3%
	Less Than 1 Hour	4	7.1%
	More Than 24 Hours	27	48.2%
	Not Applicable/ Doesn't Clean Mobile Phone	17	30.4%
Hand wash before use	No	38	67.9%
	Yes	18	32.1%
Hand wash after use	No	51	91.1%
	Yes	5	8.9%
Using a mobile phone to check time	No	18	32.1%
	Yes	38	67.9%

The majority (33.9%) of HCPs occasionally clean their mobile phones with 16.1% cleaning their phones one time per day, 3.57% cleaning their phones more than one time per day and 26.8% rarely cleaning their phones.

Among cleaning agents used by participants, dry wipe cleaning is used by the majority (57.1%) followed by alcohol and others (Clorox, lens cleaning solutions, soap water, etc.) with both around 21%. The majority (48.2%) of mobile phones collected were cleaned more than 24 hours ago followed by 30.4% of HCPs who don’t clean their phones.

The distribution of age with the gender of participants in the study population shows that the female gender has a mean age of 29 (SD 7) and the male gender has a mean age of 25 (SD 5). A total of 60.5% of doctors who participated in the study are male, 62.5% of nurses are male and 60% of other healthcare workers are female. Among participants who use their mobile phones in the hospital, 60.0% are male. Participants who clean their phones 1x/day consist of 55.5% of males and participants who clean their phones more than 1x/day consist of all males (100%). 63.6% of males never clean their phones and 68.4% of males occasionally clean their phones; participants who rarely clean their phones consist of 53.5% of females. 56.25% of participants who use dry-wipe cleaning are males; 69.23% who use alcohol are males. Participants who follow hand washing before using phones are 66.7% males and those who follow hand washing after using phones are 60% males. Participants who check their time using phones are 57.9% males and 42.1% females. The distribution of male and female participants with pre-intervention bacterial load shows that the male gender has a mean of 742.73 (SD 1440.376) and the female gender has a mean of 313.13 (SD 255.275) respectively. Independent t-test is applied between the two groups which shows statistical insignificance with a p-value of 0.164.

Table 2 shows the bacterial load, presented as colony-forming units/CFU, following intervention has drastically reduced to 27.29 ± 49.87 and 22.18 ± 33.46 in both ultraviolet radiation and disinfectant wipes groups compared before intervention bacterial load of 796.79 ± 1537.61 and 335.79 ± 360.81 respectively. Wilcoxon signed ranks test was applied between pre- and post-test groups as the data is not normally distributed both in UR and DT groups which shows statistical significance with a p-value less than 0.05.

**Table 2:** Association between pre- and post-intervention bacterial contamination

Intervention agent	Bacterial load pre-intervention	Bacterial load post-intervention	P-value
	Mean ± SD	Mean ± SD	
Ultraviolet radiation (n=28)	796.79 ± 1537.61	27.29 ± 49.87	0.00
Disinfectant wipes (n=28)	335.79 ± 360.81	22.18 ± 33.46	0.00

Mann-Whitney U test is applied in Table 3 between the two groups as the data is not normally distributed which shows a p-value of 0.071 which is greater than 0.05 and thus statistically insignificant.

**Table 3:** Association between two interventions

Variable	Intervention agent	n=56	25th percentile	50th percentile	75th percentile	P-value
Mean difference of both groups	Ultraviolet radiation	28	217	360	602	0.071
	Disinfectant wipes	28	61	223	430	

Table 4 shows the distribution of samples of the study in specific ranges of colony-forming units between two interventions in the pre-intervention bacterial load analysis and Table 5 shows the distribution of samples of the study in the post-intervention bacterial load analysis.

**Table 4:** Distribution of samples in CFU range in Pre-intervention analysis

Bacterial load (Expressed as Colony-forming units/CFU)		Intervention	
		Disinfectant wipes	Ultraviolet radiation
		N=28	N=28
Pre-intervention Bacterial load	<100 CFU	8	4
	101-500 CFU	14	12
	501-1000 CFU	4	9
	1000-5000 CFU	2	1
	>5000 CFU	0	2

**Table 5:** Distribution of samples in CFU range in post-intervention analysis

Bacterial load (Expressed as Colony-forming units/CFU)		Intervention	
		Disinfectant wipes	Ultraviolet radiation
		N=28	N=28
Post-intervention bacterial load	<100 CFU	27	26
	100-500 CFU	1	2

It is observed from Table 6 that participants using disinfectant wipes as intervention had a 2.07 times higher chance of having a low bacterial load i.e., less than 100, however, it is statistically insignificant as the p-value is more than 0.05.

**Table 6:** Analytical table showing samples in specific CFU range in post-intervention analysis and their association among two interventions.

		Post-intervention		Odds ratio (95% CI)	p-value
		<100	100-500		
Intervention	Disinfectant wipes	27 (96.4%)	1 (3.6%)	2.07 (0.17-24.31)	0.553
	Ultraviolet radiation	26 (92.9%)	2 (7.1%)		

## Discussion

The present study was conducted in a tertiary care hospital in Pondicherry to identify the pattern of mobile phone usage and the efficacy of two interventions namely ultraviolet radiation and disinfectant wipes in the decontamination of mobile phones. Out of 56 participants, 89.3% use their phones in the hospital which is less than the finding of a study by Hitti et al. [15] where it was found that 100% use their phones inside the hospital. Since HCPs use their phones for considerable time inside the hospital, which may be due to the nature of their work, in pre-intervention analysis, it was found that all 56 samples were contaminated with bacteria which is equal to what was found in a study by Tagore et al. (100%) [16] and more than that of the study conducted by Badr et al. (93.7%) [17], Dutta et al. (72%) [18]. Among the cleaning agents, the most common found to be used was dry wipes followed by alcohol and other agents. The argument for using dry wipes can be given by the fact that commercially available isopropyl alcohol can be costly against dry wipes which naturally becomes a better option in the eyes of participants in a developing country where the study was held. The use of isopropyl alcohol as a cleaning agent can significantly reduce the bacterial contamination of mobile phones but as the percentage of participants who use alcohol as a cleaning agent is less (21.4%), chances of contamination among the study population would be higher. More than 90% of participants don’t follow hand washing after phone use which is more than that of the study by Shakir et al. (47%) [19] and thus increases the cross-contamination in the hospital environment. Moreover, hand hygiene alone can significantly reduce the risk of cross-transmission of infection in healthcare facilities which was justified by Mathur in his study [20]. Among the participants who use their mobile phones in the hospital, 60.0% are males which might be due to the reason that females keep their mobiles in purses and use them less frequently during their duties. On the other hand, male doctors keep their mobiles in their pockets and use them frequently anywhere, anytime whenever it is needed, thus their mobiles are more contaminated, which was given as an argument in the study by Kokate et al. [12] to explain the high prevalence of contamination among males.

While analyzing and comparing the pre-intervention bacterial load with the post-intervention load, post-intervention bacterial contamination in terms of colony-forming units/CFU has drastically reduced after both interventions which is validated by statistical significance. So, with the use of any intervention from the mentioned interventions, bacterial load or bacterial contamination can be reduced significantly. However, while comparing the difference in efficacy between the two interventions, it was found to be statistically insignificant. Both decontamination interventions are equally effective in reducing the bacterial load and the study doesn’t necessarily point towards any single intervention.

Moreover, it was observed participants using disinfectant wipes as an intervention have a 2.07 times higher chance of having a low bacterial load i.e., less than 100 CFUs, however, it is not statistically significant. Moreover, disinfectant wipes have become a better and practically feasible method compared to hazardous ultraviolet radiation in the form of UVC lamps. Safety study results showed dermal effects of UV-C exposure, including DNA lesions, formation of cyclobutane pyrimidine dimers in cells, and effects on the skin’s stratum corneum which was given as an argument against ultraviolet radiation in the study by Ramos et al. [21].

The small sample size might have influenced the power required to reveal significant differences. The approach of the study can be modified to increase the sample size thereby eliminating limitations based on sample size. Qualitative analysis or the effect of mentioned interventions on various types of microorganisms wasn’t analysed and can open up new avenues of research for the prevention of nosocomial infections and various infectious disease control interventions during outbreaks.

## Conclusions

From this study, it can be concluded that both ultraviolet radiation in the form of UVC lamps and disinfectant wipes are effective in reducing the microbial contamination of mobile phones. Thus, any decontamination technique out of the mentioned two has a significant effect on the difference in bacterial contamination (CFU) between pre-intervention and post-intervention analysis which is depicted in the study. It was also found that male doctors have more bacterial load than females, which can be minimized by effectively changing behavioural habits. Proper hand washing should be followed before and after using mobile phones which can reduce the cross-contamination in the hospital environment. Regular cleaning of phones is also essential for decreasing microbial contamination in healthcare facilities.

It is necessary to increase awareness and change behavioral perceptions towards the use and decontamination of mobile phones in a tertiary care hospital or any other healthcare facility as an important public health initiative.
